# Changes of Brain Connectivity in the Primary Motor Cortex After Subcortical Stroke: A Multimodal Magnetic Resonance Imaging Study

**DOI:** 10.1097/MD.0000000000002579

**Published:** 2016-02-12

**Authors:** Yongxin Li, Defeng Wang, Heye Zhang, Ya Wang, Ping Wu, Hongwu Zhang, Yang Yang, Wenhua Huang

**Affiliations:** From the Institute of Clinical Anatomy, School of Basic Medical Sciences, Southern Medical University, Guangzhou (YL, YW, YY, HZ, WH); Department of Imaging and Interventional Radiology, The Chinese University of Hong Kong, Hong Kong (DW); Shenzhen Institutes of Advanced Technology, Chinese Academy of Sciences, Shenzhen (HZ); and The 3rd Teaching Hospital, Chengdu University of Traditional Chinese Medicine, Chengdu, China (PW).

## Abstract

The authors investigated the changes in connectivity networks of the bilateral primary motor cortex (M1) of subcortical stroke patients using a multimodal neuroimaging approach with antiplatelet therapy. Nineteen patients were scanned at 2 time points: before and 1 month after the treatment. The authors assessed the resting-state functional connectivity (FC) and probabilistic fiber tracking of left and right M1 of every patient, and then compared these results to the 15 healthy controls. The authors also evaluated the correlations between the neuroimaging results and clinical scores.

Compared with the controls, the patients showed a significant decrease of FC in the contralateral motor cortex before treatment, and the disrupted FC was restored after treatment. The fiber tracking results in the controls indicated that the body of the corpus callosum should be the main pathway connecting the M1 and contralateral hemispheres. All patients exhibited reduced probability of structural connectivity within this pathway before treatment and which was restored after treatment. Significant correlations were also found in these patients between the connectivity results and clinical scores, which might imply that the connectivity of M1 can be used to evaluate the motor skills in stroke patients.

These findings can help elucidate the neural mechanisms responsible for the brain connectivity recovery after stroke.

## Abstract 2

Supplemental Digital Content is available in the text

## INTRODUCTION

Ischemic stroke is one of the most common causes of mortality and morbidity in the world. It results from a focal or widespread loss of blood supply to the brain tissue, which eventually leads to a sudden disability in motor control. Previous studies have demonstrated that there is structural and functional disorder of the perilesional and remote brain regions in both hemispheres after an ischemic stroke.^1–4^ Increased activation in the primary motor cortex (M1) is highly consistent across studies in stroke patients.^5^

The role of M1 in motor recovery after stroke, however, remains controversial.^2^ The M1 is a brain region, which is located in a portion of the frontal lobe. It works in association with other motor and subcortical regions to plan and execute movements.^6^ Previous studies indicated that the contralesional M1 may play a supportive role in motor function recovery after stroke.^5,7,8^ Other studies have demonstrated that enhanced activity in the contralesional M1 may impair the recovery of motor function. The patient's motor performance of the stroke-affected hand was improved when the contralesional M1's excitability was inhibited by using transcranial magnetic stimulation.^9,10^ Hence, identifying the role of M1 during recovery processing is a current challenge in the stroke domain. Connectivity-based approaches would provide an opportunity to identify the role of M1 during recovery processing after stroke.^11^ The brain is a complex integrative network of functionally linked regions. The mapping of abnormal brain networks is a popular recent effort in the study of brain dysfunction.^12^ Previous studies mainly focused on the brain changes occurring after a stroke.^2,13,14^ The treatment effect on the brain's activation and white matter (WM) integrity were also investigated after a stroke.^15,16^ Little attention, however, has been paid to the changes of the brain's functional and structural connectivity with respect to antiplatelet therapy after a stroke. Investigation into the reorganization of M1 networks after a stroke may provide additional understanding of the mechanisms underlying recovery processing.^17^

Functional and structural connectivity analyses are the 2 main approaches used presently to study brain organization. Functional connectivity (FC) networks are derived from estimates of interactions among a time series of neuronal activity. Resting-state functional magnetic resonance imaging (fMRI) is a widely used method from which the FC of the brain can be measured. Structural connectivity networks represent anatomic wiring diagrams. Diffusion tensor imaging (DTI) is another advanced technique, which can be used to probe and quantitatively evaluate the structural connectivity of WM tracts. Combining both resting-state fMRI and DTI methods, it is possible to take advantage of complementary views on brain connectivity after strokes to enhance our understanding about the neural information processing reflected in each modality.

Therefore, in this study, we used the resting-state fMRI and DTI methods simultaneously to examine the brain connectivity responsible for treatment-induced recovery after stroke. We hypothesized that functional and structural connectivity abnormalities between the hemispheres in patients with subcortical stroke would be restored after clinical treatment. Bilateral M1 were selected to investigate the changes in connectivity networks of stroke patients before and after clinical treatment to identify the role of M1 during the recovery processing with treatment after a subcortical stroke. In addition, stroke commonly causes a loss of motor function. Previous studies have found that changes in brain connectivity of stroke patients relate to impaired motor behavioral and functional recovery.^2,16,18^ The structural integrity of the transcallosal tracts is a relevant factor influencing functional recovery after stroke.^19^ Hence, we furthermore hypothesized that brain connectivity between the hemispheres would be predicated by patient's motor skills and daily living abilities. To test this hypothesis, the clinical scores, such as Fugl-Meyer Motor Assessment (FMA) and Modified Barthel Index (MBI), were assessed before and after treatment in the stroke patients. Correlations between the brain connectivity and the clinical scores were investigated, which may provide additional information for useful in explaining the connectivity changes and understanding the brain rewiring after strokes.

## MATERIALS AND METHODS

### Subjects

A total of 19 adults (8 women; mean 64.7 years) diagnosed with unimanual motor deficits because of subcortical ischemic lesions were included from Department of Neurology of the First Affiliated Hospital of Chengdu University of Traditional Chinese Medicine in China. The inclusion criteria were as follows: first-ever ischemic stroke with strictly subcortical lesions and absence of other WM pathology as verified by structural MRI; no previous or subsequent cerebral ischemia; no additional psychiatric or neurologic disorders; time after stroke onset of >20 days; right-handness before stroke; absence of neglect, aphasia, and dementia. No other experimental therapy was performed on any patient before enrolling into this study. All the patients were scanned using resting-state fMRI and DTI (diffusion data of 6 patients were not performed) at 2 time points: before and 1 month after antiplatelet therapy. We assessed the clinical scores of these patients before and after treatment using FMA and MBI. The control group consisted of 15 healthy volunteers (6 women; mean 62.1 years) with no history of neurologic or psychiatric disorders. Participants in the control group had no significant difference from the patient group with respect to age or sex. All participants in the control group were only scanned once: on the day when they were recruited for this study. Information on both groups can be found in Table 1. The experiment was approved by the Ethics Committee of Chengdu University of Traditional Chinese Medicine, and the method was carried out in accordance with the approved guidelines. Written informed consent was obtained from each subject before the study.

**TABLE 1:**
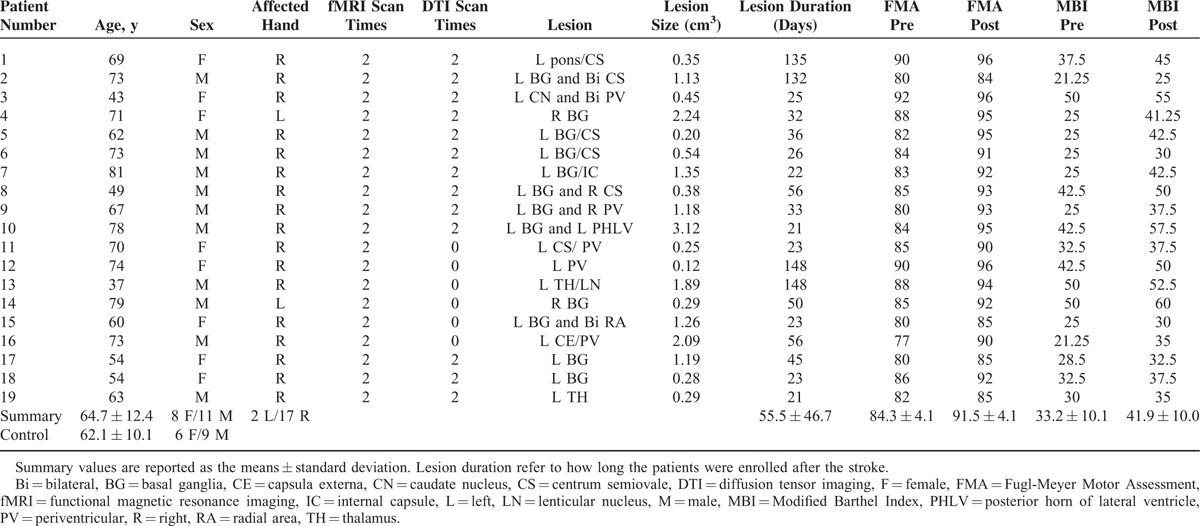
Demographic and Imaging Data

### Treatment and Clinical Assessments

All patients were given an antiplatelet therapy: enteric-coated aspirin (100 mg, daily) was used to inhibit platelet aggregation. Then citicoline (0.5 g, daily) was injected intravenously to improve the clinical outcome following an ischemic stroke. The clinical scores (FMA and MBI) were assessed on the day when patients were tested with the diffusion/functional MRI. The FMA is a well-designed, feasible, and efficient clinical examination approach that has been widely used in stroke populations.^20^ The higher scores of FMA denote milder impairments in motor function. A 10 question, 100-point version of the MBI was used to indicate the daily living abilities achieved by the patients. The MBI is frequently used in clinical practice and as a trial outcome measure in strokes.^21,22^ A paired *t* test was conducted to determine whether the stroke patients had actually improved in terms of clinical scores from pre- to posttreatment. Differences in clinical scores from pre- to posttreatment were calculated as proportional change: (post–pre)/pre × 100. The simple correlation between the proportional change of clinical scores and lesion sizes was calculated for the patient group. A similar correlation between the proportional change of clinical scores and lesion duration was also calculated.

### Image Acquisition

Magnetic resonance images were acquired using a 3T Siemens scanner (MAGNETOM Trio Tim, Siemens, Erlangen, Germany) at the West China Hospital MRI Center, Chengdu, China. During the examination, foam cushions were used in the scan process to reduce head translation movement and rotation. Resting-state fMRI was collected using an echo-planar imaging sequence with the following scan parameters: repetition time = 2 seconds, echo time = 30 milliseconds, field-of-view = 240 mm × 240 mm, matrix = 64 × 64, flip angle = 90°, slice thickness = 5 mm, 30 interleaved axial slices, and 180 volumes. All participants were instructed to keep their eyes closed and to remain motionless.

Thirteen stroke patients (5 women; mean 64.4 years) underwent the DTI scan in this study. Diffusion MRI data were collected using a spin-echo planar imaging sequence with the following parameters: repetition time = 6.8 seconds, echo time = 93 milliseconds, number of excitation = 2, flip angle = 90°, field-of-view = 240 mm × 240 mm, in plane resolution = 1.875 × 1.875 mm^2^, 50 axial slices, slice thickness = 3 mm, 30 noncollinear diffusion directions with a b-value of 1000 seconds/mm^2^, and 1 with a b-value of 0 seconds/mm^2^. The images that were not diffusion-weighted images served as an anatomic reference for motion correction.

### Imaging Processing and Statistical Analysis

#### Functional Connectivity Analysis

**FIGURE 1:**
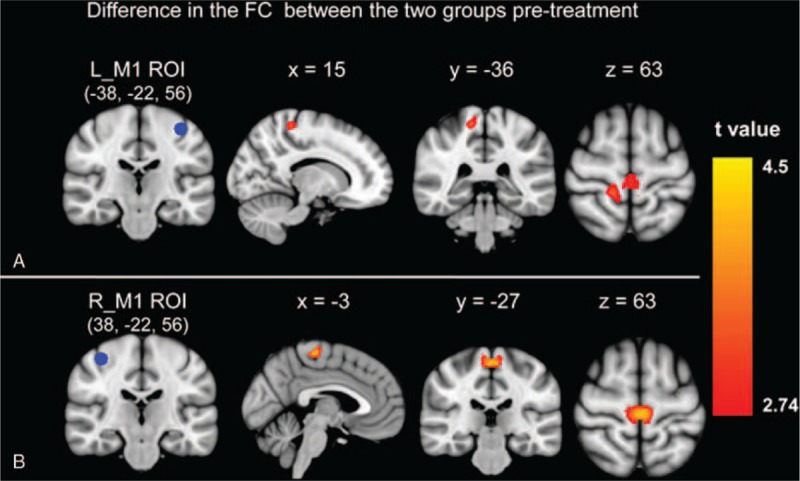
Group differences in the functional connectivity from left/right M1 between the stroke patients pretreatment and the controls. The significant group differences between functional connectivity of left M1 region of interest (A) and right M1 region of interest (B) on the rest of the brain. M1 = primary motor cortex.

The resting-state fMRI data were processed using the statistical parametric mapping (SPM8, London, UK, http://www.fil.ion.ucl.ac.uk/spm) package. The preprocessing steps included slice timing, spatial realignment, normalization into the Montreal Neurological Institute template, and smoothing. A temporal filter (0.01–0.08 Hz) was then applied and nuisance regression was also performed using WM, cerebrospinal fluid, and the 6 head motion parameters as covariates. Whole-brain functional connectivity maps were generated using the voxel-wise approach by computing the FC values between the region of interest (ROI) and each voxel within the brain were computed. Two sphere ROIs (radius = 6 mm) were created according to Wang et al^23^: the left M1 (x = −38; y = −22; and z = 56) and right M1 (x = 38; y = −22; and z = 56) (left panel of Figure 1). Finally, 2-sample *t* tests were performed to find areas that showed significant differences in FC between participants with stroke and the controls both before and after treatment. The significantly different regions between patients and the controls were selected and the FC values were extracted for each participant. Partial correlation analyses were computed between patients’ clinical scores and the FC values. Age, sex, lesion size, and lesion duration were controlled as covariates in all the above statistical analyses. Analysis details were in the accomplished through Supplement Information Methods.

#### Probabilistic Fiber Tracking

To better understand the spatial location and connectivity of the WM fibers identified by the bilateral M1, the probabilistic fiber tracking method was used.^24^ The DTI data were analyzed using the FMRIB software library, London, UK (FSL, v5.0.2, www.fmrib.ox.ac.uk/fsl). The images were initially corrected for eddy current distortion and head motion, and subsequently a diffusion tensor model was fitted at each voxel of the corrected data with DTIFIT. The ROIs were linearly transformed into the native space of each participant. For each participant, the axial diffusivity (λ//, AD), radial diffusivity (λ⊥, RD), mean diffusivity (MD), and Fractional anisotropy (FA) were calculated. Then, fiber tracking was performed using a probabilistic tractography algorithm implemented in FSL (probtrackx). The 2 sphere ROIs for tractography were defined similarly in the same way as used in the FC analyses.

The tracking results for each participant were then thresholded and summed across subjects to produce group probability maps (pretreatment map, posttreatment map, and control map) for each pathway. These group probability maps were thresholded again and masked with the corpus callosum (CC) to produce transcallosal maps. Mean diffusion indices (FA, AD, RD, and MD; only nonzero values) in the transcallosal maps were calculated for each participant. Partial correlation analyses between these mean diffusion indices and clinical scores were calculated. Analysis details were in the SI Methods.

## RESULTS

### Behavioral Measures

Information on all subjects is listed in Table 1. Fugl-Meyer Motor Assessment scores changed (pair T: *t* = −10.077, *P* < 0.001) from 84.3 ± 4.3 at the baseline measure (pretreatment) to 91.5 ± 4.1 after the treatment (posttreatment). The stroke patients showed significant enhancement of their motor skills as measured by FMA 1 month after the treatment intervention. The MBI scores changed significantly from 33.2 ± 10.1 at the baseline measure to 41.9 ± 10.0 after the treatment. The stroke patients showed significant improvement in the degree of their daily living abilities as measured by the MBI from pre- to posttreatment (pair T: *t* = −7.461, *P* < 0.001). No association was found between the proportional change of clinical scores (FMA and MBI) and lesion parameter (lesion size and duration).

### Functional Connectivity Results

For the left M1 ROI, the stroke patients before treatment showed reduced connectivity with the right postcentral and bilateral paracentral cortex as compared with the controls (Figure 1A). For the right M1 ROI, stroke patients before treatment showed reduced connectivity with the bilateral paracentral cortex as compared with the controls (Figure 1B). No greater connectivity was found for patients before treatment as compared with the controls for either the left or right M1 FC results. There was no longer a significant difference in the connectivity for either the left or right M1 between the patient group and the controls after treatment. Table 2 provides a summary of the FC results.

**TABLE 2:**
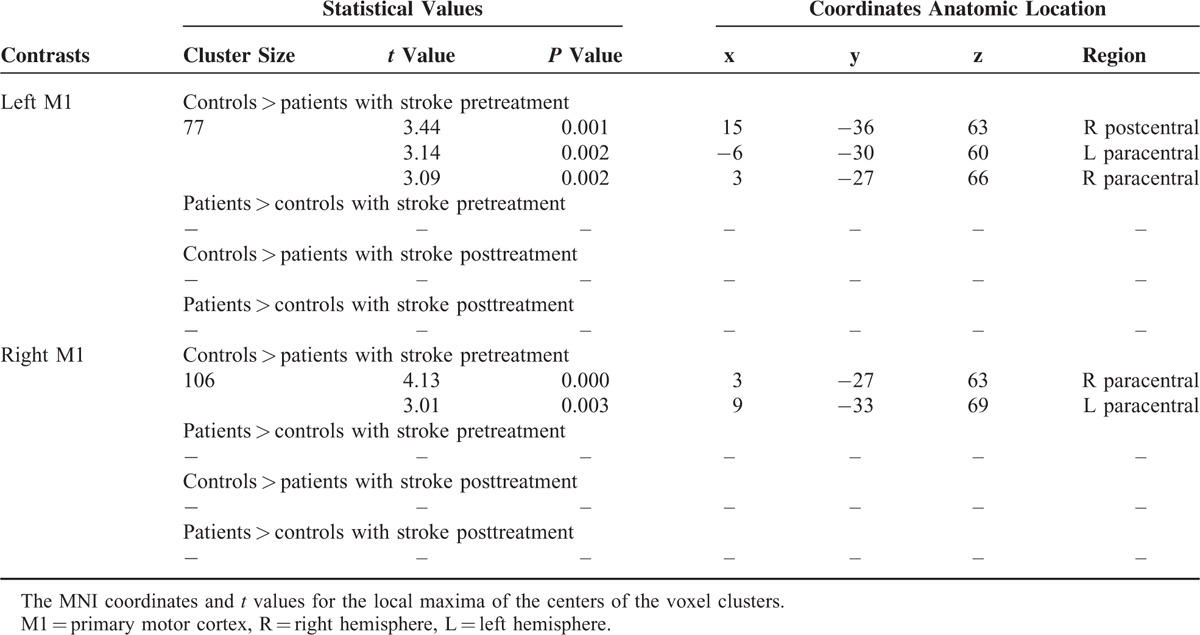
Two-sample *t* Test of the Difference in the Functional Connectivity of Bilateral Primary Motor Cortex With the Rest Brain Between Patients and Controls

**FIGURE 2:**
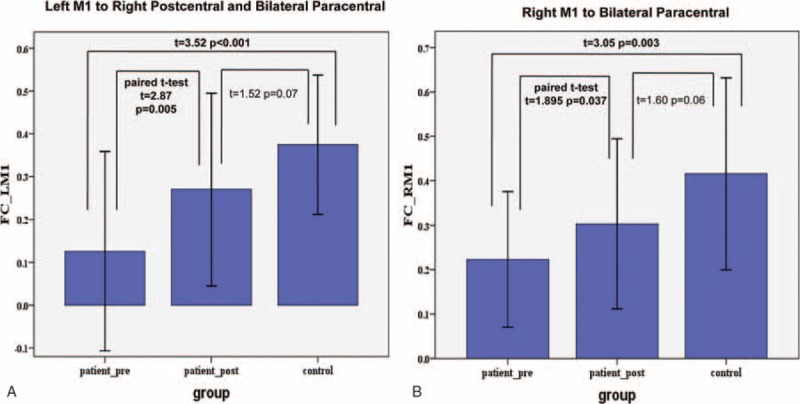
Group comparisons of resting-state functional connectivity between groups. Bars represent the mean and error bars represent the standard deviations.

Functional connectivity analyses results revealed that stroke patients had decreased connectivity as compared with the controls (Figure 2). Figure 2A showed an increased FC of the left M1 from pre- to posttreatment (*P* = 0.005; 1-tailed). Figure 2B showed a significant FC changes of the right M1 from pre- to posttreatment (*P* = 0. 037; 1-tailed).

The partial correlation analysis results showed in Table 3. There were significant positive correlations between the clinical scores and the mean FC values extracting from left M1 FC results (FMA versus FC values: *r* = 0.323, *P* = 0.031; MBI versus FC values: *r* = 0.427, *P* = 0.006; 1-tailed). There were similar correlations between the clinical scores and the mean FC values from right M1 FC results (FMA versus FC values: *r* = 0.350, *P* = 0.021; MBI versus FC values: *r* = 0.408, *P* = 0.009; 1-tailed).

**TABLE 3:**
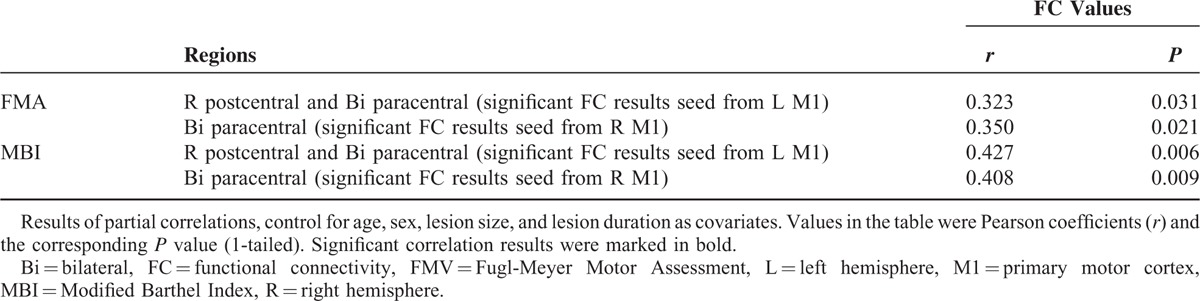
Partial Correlation Analyses Between the Functional Connectivity Results and Fugl-Meyer Motor Assessment Scores

### Probabilistic Fiber Tracking Results

**FIGURE 3:**
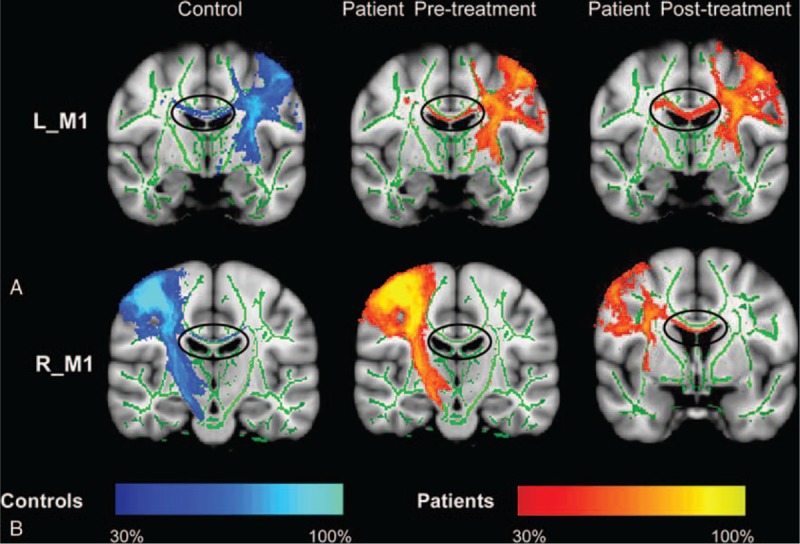
Probabilistic fiber tracking with seed in the left/right primary motor cortex. The body of the corpus callosum was the main pathway connecting the seeds to the contralateral hemisphere. The tracts in stroke patients showed a structural connectivity changed from pre- to posttreatment.

The tracking results (Figure 3) showed that the body of the CC and corticospinal tracts were the principle fiber pathways connecting left/right M1 in the control group. Patients exhibited a reduced probability of connectivity between the left M1 and right hemisphere before treatment and the disrupted pathway was restored in the body of the CC after treatment (Figure 3A). Similar tracking results were found to be seeded in the right M1 of the patient group (Figure 3B).

Table 4 shows the relationship between the diffusion indices and clinical scores for the stroke patients group. For the transcallosal tracts from the left M1 to the right hemisphere, partial correlation analyses of tract-specific mean FA with clinical score yielded significant or near significant results (FA versus FMA: *r* = 0.314, *P* = 0.078; FA versus MBI: *r* = 0.385, *P* = 0.039; 1-tailed). Partial correlative analyses indicated a significantly negative relationship between RD/MD and the clinical score (MD versus FMA: *r* = −0.430, *P* = 0.023; RD versus FMA: *r* = −0.407, *P* = 0.030; MD versus MBI: *r* = −0.368, *P* = 0.046; and RD versus MBI: *r* = −0.419, *P* = 0.026; 1-tailed). A trend negative relationship was found between the FMA and AD. No significant correlation was found between the MBI and the AD.

**TABLE 4:**
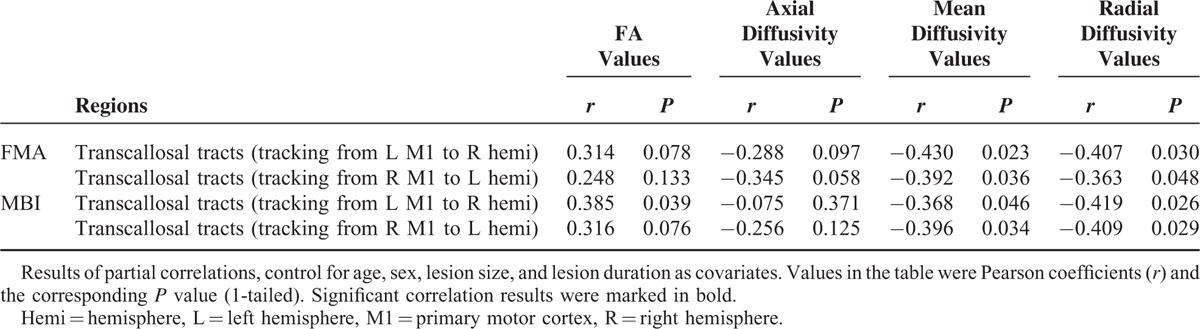
Partial Correlation Analyses Between the Fiber Tracking Results and Fugl-Meyer Motor Assessment Scores

For the transcallosal tracts from right M1 to the left hemisphere, partial correlation analyses of tract-specific mean RD/MD with clinical score yielded significant results (MD versus FMA: *r* = −0.392, *P* = 0.036; RD versus FMA: *r* = −0.363, *P* = 0.049; MD versus MBI: *r* = −0.396, *P* = 0.034; and RD versus MBI: *r* = −0.409, *P* = 0.029; 1-tailed). Partial correlative analyses indicated a negative trend relationship between the FA and the clinical score. A positive trend relationship was found between the AD and the FMA, but not found between the AD and the MBI.

## DISCUSSION

The brain is organized into a set of widely distributed networks. The mapping of abnormal brain networks is currently a popular framework for the study of brain dysfunction. In the current study, we investigated the differences in the brain connectivity networks between healthy subjects and subcortical stroke patients in both the pre- and the posttreatment stages. In addition, we observed the correlations between the brain connectivity results and the clinical scores. Our results demonstrated that connectivity analyses of M1 may provide new insights into the neural mechanisms responsible for the brain recovery after strokes.

### Brain Changes in Functional Connectivity

In current study, the recovery process of interhemispheric FC after strokes was consistent with previous findings in animal and human studies.^7,8,25^ Previous research on recovery processing after strokes in rats showed that interhemispheric connectivity between the ipsilesional primary sensorimotor cortex and the contralesional sensorimotor cortex was significantly diminished during the first few days.^25^ The diminished connectivity subsequently regained with sensorimotor functions recovery. In human, longitudinal changes of resting-state FC in ipsilesional M1 were assessed during motor recovery after strokes.^7^ The FC between the ipsilesional and contralesional M1 in stroke patients showed the most asymmetry at 1 month after onset and then restored the interhemispheric functional coherence at 6 months after onset. Similarly, dynamic changing processes were found in motor-related areas during a motor task activation study.^5^ Neural activity is often enhanced in motor-related areas in both hemispheres shortly after ischemic strokes; returning to levels similar to those observed in healthy subjects over 12 months after stroke onset. These previous observations lead us to speculate that the network connecting the bilateral M1 would change with the recovery process after a stroke. Our FC results support this notion and indicate that the reinstatement of FC in the bilateral M1 is an important feature of recovery after stroke.

Furthermore, we focused our attention on the treatment effects upon stroke patients with motor disorder. The experimental design in the current study was different from the previous studies (listed above). We used enteric-coated aspirin drug combining with citicoline injection as an intervention means to study the recovery process after strokes. All patients received this clinical treatment for 1 month. Patients diagnosed with stroke showed a reduced FC between M1 and the contralateral motor cortex before treatment. These FC results supported the fact that functional deficits can extend to remotely connected areas.^23,26,27^ After treatment, the disrupted FC was restored. In previous neuroimaging studies, the brain FC and the activation of the bilateral M1 in patients was restored within 6 to 12 months after a stroke onset.^5,7^ Compared with these previous studies, we can see that treatment intervention may accelerate the recovery process in stroke patients. The restored FC can be detected as early as 1 month after treatment. The neuroimaging results of the current study pointed out that antiplatelet therapy is a main factor during the recovery process after a stroke. Our FC results were also supported by the behavioral observations in the current study. At a behavioral level, the motor skills and daily living abilities in stroke patients were improved immediately after treatment. The changes in behavioral and resting-state FC were in accordance with each other.

### Brain Changes in Structural Connectivity

In our study, fiber tracking results revealed that the body of the CC was the main pathway connecting the M1 and contralateral hemispheric regions in the controls. This result can be explained by the relationship between the brain's functional and structural networks. Structural connectivity is defined as the existence of WM tracts physically interconnecting brain regions. Previous studies found that resting-state connectivity was shown to be related to structural connectivity.^28^ In the current study, the resting-state FC analyses indicated that the FC of bilateral M1 was higher in the controls. So, it is noteworthy to expect that there is a strong similarity between the fiber tracking maps and the resting-state FC maps. Our fiber tracking results were consistent with this expectation. Tracts in the body of the CC were detected as the main pathway connecting the M1 and the contralateral motor regions in the controls. As we known, the CC is the largest WM structure in the brain, connecting the homologous cortical areas of the 2 cerebral hemispheres. The midbody of the CC contains the commissural corticocortical fibers that connect bilateral sensorimotor and premotor cortex.^29–31^ Increased integrity in the commissural fibers may relate to the increased functional activity in these motor cortical areas or to the increased FC between these motor areas. Fiber tracking of the transcallosal pathway was successful in the controls, which was consistent with prior diffusion studies.^29,31,32^ Our results provide evidence that the middle part of the CC is connected to the bilateral motor areas.

Stroke patients exhibited a reduced probability of connectivity within the body of the CC before treatment and which was restored after treatment. This result can be explained by the important role of the transcallosal fibers in the stroke recovery process. Recent diffusion MRI studies revealed that stroke could induce WM abnormalities in the CC.^3,16,19,32^ Degeneration of transcallosal fibers can be used to predict the functional motor potentials in chronic stroke patients^16^ and the recovery of motor functions after stroke.^19^ Interhemispheric interaction models have shown that transcallosal connections play an important role during stroke recovery.^30^ In the current study, the observed reduction of probability connectivity in the transcallosal pathway can be attributed to the strokes effect. After a stroke, the integrity of myelin sheath was lost along the body of the CC. With treatment intervention, WM remodeling occurred in this tract. So the probability of connectivity within the body of the CC was improved with treatment after stroke.

The main research focus of our current study is to detect the longitudinal changes of brain connectivity in strokes patients with treatment. Although longitudinal changes of FC have been studied during motor recovery after stroke in previous studies,^7,8,33^ only a few studies have assessed the longitudinal changes of structural connectivity during the stroke recovery process.^32^ Our study demonstrated that the microstructure of the body of the CC may change during the period of treatment after stroke. We can see that the changes in FC were demonstrated to consistently change with structural connectivity. Recovery process with treatment of stroke patients may affect both the functional and structural connectivity of the motor networks. These results provide further support for a structural connectivity mechanism underlying changes in FC.^25,34^ Based on these findings, we propose that cortical remapping of functions after a stroke may have some association with changes in structural connectivity. The disrupted functional and structural networks might restore with treatments after stroke.

### Correlations Between Connectivity Results and Clinical Scores

In this study, we showed that there were significantly positive correlations between the resting-state FC results and patients’ clinical scores. This result is consistent with several other stroke studies that demonstrated significant correlation between behavioral deficits and changes in FC.^2,18,25,35^ The bilateral M1 regions were the main ROIs in most of these studies. For the ipsilesional M1, a previous study showed that the FC of this region with the contralesional supplementary motor area and middle frontal gyrus at onset was positively correlated with the motor recovery performance at 6 months after a stroke.^7^ Our FC findings were compatible with this result showing that FC of the ipsilesional M1 can be used to predict motor skills. In the contralesional M1, the functional role of this region has also been demonstrated in stroke patients. Longitudinal resting-state fMRI showed that the dynamic change in the interhemispheric FC of the contralesional primary sensorimotor cortex in subcortical stroke patients was positively correlated with the Motricity Index scores.^8^ These reports, together with our FC findings, suggest that stroke leads to the changes of interhemispheric FC particularly between the motor areas of both hemispheres. The interhemispheric FC changes can be expected by the motor skills and daily living abilities of stroke patients. Our FC results provide further evidence for the supportive role played by the bilateral M1 in motor function recovery after stroke.

In addition, a correlation analysis showed that the diffusion indices (such as MD and RD) of the body of the CC were significantly correlated with patients’ motor skills and daily living abilities. A significantly positive relationship was also found between the mean FA value of the body of the CC and the clinical scores. This structural–behavioral correlation result was consistent with our expectation. Previous DTI studies have demonstrated the crucial role of the CC after stroke.^3,19,36,37^ The body of the CC may be involved in the recovery of motor function in stroke patients.^19,32^ A previous study in subcortical stroke patients showed that the FA values of clusters in the midbody of the CC were positively correlated with the recovery of motor function.^32^ Lower FA in the CC was associated with poor performance in the action research arm test of stroke patients.^19^ Thus, we expected that the patient with a higher integrity of the CC would show more potential for the recovery of cognitive function, such as enhancing their motor skills and their daily living abilities. Our partial correlation analysis results showed that poor FMA and MBI performance were associated with both lower FA and higher direction indices (RD and MD) in the body of the CC. These correlation results suggested that the structural integrity of the body of the CC is a relevant factor influencing behavioral recovery after stroke. In the current study, a trend positive relationship was also found between the AD and the FMA, but was not found between the AD and the MBI. The reason for this performance may be that the scale sensitivity in detecting the motor function was different between the FMA and MBI. Previous DTI studies in chronic stroke patients showed that the transcallosal tract diffusivity had the greatest predictive value of functional recovery. Greater gains in motor function were related to lower AD and RD of the transcallosal tracts.^16^ The FMA is a well-designed, feasible, and efficient clinical examination approach to evaluate stroke patient's motor skills, especially the limb movement.^20^ The MBI is one of the most widely used measures of self-care performance in rehabilitation of people with stroke.^21,22^ Although the MBI can partly reflect the patient's motion function, its measure is not limited to limb movement. So the correlations between the AD and MBI were not detected.

### The Mechanism Underlying These Connectivity Changes

Compared with the controls, the stroke patients showed a significant decrease of FC in the contralateral motor cortex before treatment, and the disrupted FC was restored after treatment. For the structural connectivity, all stroke patients exhibited reduced probability of structural connectivity within the pathway connecting the M1 and contralateral hemispheres before treatment and which was restored after treatment. The reason for these connectivity changes can eliminate the factor of spontaneous recovery. Previous study have showed that the largest improvements occurs in the first 30 days after stroke of the early spontaneous recovery.^38^ Study on arm disability revealed that a maximum of function is reached by 80% of the patients within 3 weeks.^39^ In our study, the illness duration of all stroke patients were more than 3 weeks at the time point for the first MRI scanning (see Table 1). We can see that patient's spontaneous recovery process is not the main factor inducing the brain connectivity changes during the intervention processing. Age and sex were matched between the both groups. So, we speculated that the brain connectivity changes might be caused by the treatment intervention. Vascular autoregulation mechanism would explain this phenomenon. Antiplatelet therapy can promote vessel recanalization and establish the flow to the ischemic areas of the brain. With this therapy, the stroke patient's functional outcomes were improved. In neuroimaging, the structural reorganization would recover between the motor-related areas with the intervention. Previous studies have showed that the flow of synaptic events through resting-state networks is constrained by underlying structural connectivity.^35,40^ In the current study, the function connectivity between the bilateral M1 was recovered with the intervention.

## LIMITATIONS

This study has several limitations. First, the experimental design needs to be improved with considering the case-controlled fashion and the collecting time points. An untreated group of stroke patients is needed in the future studies to study the effect of treatment. Stroke patients’ data were only collected at 2 time points: pretreatment and 1-month posttreatment. Previous longitudinal studies showed that the neural activity and FC of motor-related areas was found to return to normal levels over a 12-month period after stroke onset.^5,8^ In the current study, it is not possible to establish whether the alternations in brain connectivity after stroke are retainable for a long period. Future studies with multiple time points are needed to assess the brain connectivity changes in patients with stroke. Second, the sample size of current study is relatively small, especially the diffusion data. Six patients’ DTI data were not collected. This limitation reduces the statistical significance of the results. Although the FC and fiber tracking results were consistent with each other perfectly, more work is needed in this field. Further studies with more subjects will be important in providing additional support for our conclusion. Third, the lesion location in the current study is no subdivided strictly. Lesion location rather than the mere size of the lesion accounts for the motor deficit after stroke. Although we have limited the patients with subcortical lesions strictly, the subcortical areas are broad. Further studies with subdivided lesion location would be needed.

## CONCLUSIONS

The findings of the current study demonstrated that participants with stroke showed a significant decrease of FC in the contralateral motor cortex (including the postcentral and paracentral) before treatment and the decreased FC was then restored after treatment. In addition, probabilistic tractography results showed that patients exhibited a similar change tendency within the body of the CC pathway. This multimodal neuroimaging study implied that stroke could induce brain abnormalities in both the functional and structural networks. These disrupted motor networks would be restored with antiplatelet therapy after strokes. Significant correlation between connectivity results and clinical scores implied that the connectivity of M1 could be used to predict stroke patients’ motor skills and their daily living abilities. The results of the current study broaden our view that the bilateral M1 plays an important role during stroke recovery. This study may aid in understanding the neural mechanisms underlying the rehabilitation of brain connectivity after stroke.

## Supplementary Material

Supplemental Digital Content
