# Supplementary material for: Changes of Brain Connectivity in the Primary Motor Cortex After Subcortical Stroke: A Multimodal Magnetic Resonance Imaging Study
Source: Medicine (Baltimore). 2016 Feb 12;95(6):e2579. doi: 10.1097/MD.0000000000002579 (PMC4753872; doi:10.1097/MD.0000000000002579)
Supplement: Supplemental Digital Content [file medi-95-e2579-s001.doc]

**Methods**

**Imaging Processing and Statistical Analysis**

**Functional Connectivity Analysis**

Before fMRI data preprocessing, we flipped the imaging data from right to left along the midsagittal line for the two patients who had lesions on right hemisphere. The fMRI data from control group matched to these patients (who were also midsagittally oriented). The resting-state fMRI data were processed using Statistical Parametric Mapping (SPM8, http://www.fil.ion.ucl.ac.uk/spm) package. The first ten volumes of the functional images were discarded to allow for signal equilibrium. The remaining 170 volumes were slice-time corrected, realigned to the first image. The head motion parameters were estimated. One control subject’s fMRI were excluded from the analysis because of excessive head motion (which exceeded 2 mm in transition or 2^0^ in rotation). All of the realigned images were spatially normalized to the MNI template, and each voxel was resampled to 3 × 3 × 3 mm^3^. After normalization, the images were smoothed using a Gaussian kernel of 8 × 8 × 8 mm^3^ full-width at half-maximum. A temporal filter (0.01–0.08 Hz) was then applied to reduce low-frequency drifts and high-frequency physiological noise. Nuisance regression was also performed using white matter, cerebrospinal fluid, and the six head motion parameters as covariates.

Functional connectivity maps were obtained using the voxel-wise approach by computing FC between the region of interest (ROI) and each voxel within the brain. We defined two sphere ROIs (radius = 6 mm) according to Wang et al.[^1^](#_ENREF_1): the left M1 (x: -38; y: -22; z: 56) and right M1 (x: 38; y: -22; z: 56) (left panel of Figure 1). Finally, the correlation coefficients in each voxel were transformed to z values by using the Fisher r-to-z transformation to improve normality. The z-FC maps from each participant were analyzed using a separate one-sample t-test for the entire sample (controls, stroke patients both pre- and post treatment) with an FEW corrected p < 0.05 for positive coefficient. For the one-sample t-test results, we could define the search volume masks for the FC to constrain the subsequent between-group analyses. Two-sample t-tests were conducted to compare z-FC maps between participants with stroke and the controls both before and after treatment. Corrected cluster thresholds were determined using Monte Carlo simulations with the program AlphaSim in AFNI, implemented in the REST toolbox[^2^](#_ENREF_2). Using a cluster connectivity criterion of 5 mm (edge connected), a spatial smoothness of 8 mm, and a height threshold of p < 0.005, family wise error rates of p < 0.05 were achieved with a minimum cluster threshold of 46 contiguous voxels for the FC analyses. The locations of the peak maxima of significant clusters were determined using xjview toolbox (<http://www.alivelearn.net/xjview>). The significantly different regions between patients and the controls were selected and the FC values were extracted for each participant. Using SPSS 16.0 for Windows, partial correlation analyses were computed between patients’ clinical scores and the FC values. Correlations were considered significant if their p value was less than 0.05. Age, sex, lesion size and lesion duration were controlled as covariates in all the above statistical analyses.

**Probabilistic Fiber Tracking**

To better understand the spatial location and connectivity of the WM fibers identified by the bilateral M1, the probabilistic fiber tracking method was used[^3^](#_ENREF_3). Before DTI data preprocessing, we flipped the imaging data from right to left along the midsagittal line for the one patient who had lesions on the right hemisphere. The DTI data from the controls matched to this patient was also midsagittally oriented. The DTI data was analyzed using the FMRIB Software Library (FSL, v5.0.2, [www.fmrib.ox.ac.uk/fsl](http://www.fmrib.ax.ac.uk/fsl)). Motion and eddy current correction were carried out using affine registration to the first non-diffusion weighted image[^4^](#_ENREF_4). The data were then skull-stripped using the FMRIB Brain Extraction Tool (BET v2.1)[^5^](#_ENREF_5). Subsequently, the FMRIB Diffusion Toolbox (FDT v3.0) was used to fit the diffusion tensor and calculate the eigenvector and eigenvalue (λ1, λ2 andλ3) at each voxel[^6^](#_ENREF_6). Fiber tracking was performed using a probabilistic tractography algorithm implemented in FSL (probtrackx), which is based on Bayesian estimation of diffusion parameters (Bedpostx). Fiber tracking was initiated from all voxels within the seed mask in the diffusion space to generate 5000 streamline samples, with a step length of 0.5 mm, a curvature threshold of 0.2, and a maximum of 2000 steps. The two sphere ROIs for tractography were defined similarly as we used in FC analyses. The ROIs were linearly transformed into the native space of each participant. For each participant, the axial diffusivity (λ//, AD) was computed as the largest eigenvalue, and the radial diffusivity (λ⊥, RD) was defined as the average of the minor eigenvalues. A directionally averaged measure of diffusion was measured as the mean diffusivity (MD). FA was calculated from the standard deviation of the three eigenvalues ranging from 0 to 1[^8^](#_ENREF_8).

After all tracts were calculated for each participant, the tracking results were then thresholded to include only those voxels that had at least 50 samples passing through them (out of 5000 generated from each seed voxel). For each participant, the two pathways (left M1 to all brain and right M1 to all brain) were binarized, transformed to the MNI 152 brain standard space. Then the transformed images were summed across subjects to produce group probability maps (pre-treatment map, post-treatment map and control’s map) for each pathway. These group probability maps were thresholded to display paths that were present in a minimum of one-third of participants in each group. The corpus callosum (CC) mask was created from the TCBM-DTI-81 parcellation map atlas. For each pathway, these thresholded group probability maps were masked with the CC mask. The tracts inside in this mask were retained and summed across all groups to produce two transcallosal maps (transcallosal tracts from left/right M1 to contralateral hemisphere). Mean diffusion indices (FA, AD, RD, and MD; only non-zero values) in both transcallosal maps were calculated for each participant. Partial correlation between these mean diffusion indices and clinical scores were calculated. To minimize the potential impact on the findings, age, gender, lesion size and lesion duration were used as covariates of no interest in all statistical analyses described above. TCBM-DTI-81 parcellation map atlas and JHU WM tractography atlas were used to determine the location of the tract result[^9^](#_ENREF_9).

References

1. Wang L, Yu C, Chen H, et al. Dynamic functional reorganization of the motor execution network after stroke. *Brain.* Apr 2010;133(Pt 4):1224-1238.

2. Song X-W, Dong Z-Y, Long X-Y, et al. REST: a toolkit for resting-state functional magnetic resonance imaging data processing. *PloS one.* 2011;6(9):e25031.

3. Behrens TE, Berg HJ, Jbabdi S, Rushworth MF, Woolrich MW. Probabilistic diffusion tractography with multiple fibre orientations: What can we gain? *Neuroimage.* Jan 1 2007;34(1):144-155.

4. Jenkinson M, Smith S. A global optimisation method for robust affine registration of brain images. *Medical image analysis.* Jun 2001;5(2):143-156.

5. Smith SM. Fast robust automated brain extraction. *Human brain mapping.* Nov 2002;17(3):143-155.

6. Basser PJ, Pierpaoli C. Microstructural and physiological features of tissues elucidated by quantitative-diffusion-tensor MRI. *Journal of magnetic resonance. Series B.* Jun 1996;111(3):209-219.

7. Behrens TE, Woolrich MW, Jenkinson M, et al. Characterization and propagation of uncertainty in diffusion-weighted MR imaging. *Magnetic Resonance in Medicine.* Nov 2003;50(5):1077-1088.

8. Basser PJ, Mattiello J, LeBihan D. Estimation of the effective self-diffusion tensor from the NMR spin echo. *Journal of magnetic resonance. Series B.* Mar 1994;103(3):247-254.

9. Mori S, Oishi K, Jiang H, et al. Stereotaxic white matter atlas based on diffusion tensor imaging in an ICBM template. *Neuroimage.* Apr 1 2008;40(2):570-582.
